# Development of a Bicycle Road Surface Roughness and Risk Assessment Method Using Smartphone Sensor Technology

**DOI:** 10.3390/s25113520

**Published:** 2025-06-03

**Authors:** Dong-youn Lee, Ho-jun Yoo, Jae-yong Lee, Gyeong-ok Jeong

**Affiliations:** 1 Department of Road Transport, Korea Transport Institute, Sejong-si 30147, Republic of Korea; dyoun87@koti.re.kr (D.-y.L.); urbanj@koti.re.kr (G.-o.J.); 2 Research Institute, RoadKorea Inc., Yongin-si 18471, Republic of Korea; 3 Department of PPP Infrastructure Management, Korea Transport Institute, Sejong-si 30147, Republic of Korea; lbs8180@koti.re.kr

**Keywords:** bicycle road roughness index (BRI), faulting impact index (FII), surface roughness, smartphone sensor technology

## Abstract

Surface roughness is a key factor influencing the safety, comfort, and overall quality of bicycle lanes, which are increasingly integrated into urban transportation systems worldwide. This study aims to assess and quantify the roughness of bicycle lanes in Sejong City, Republic of Korea, by utilizing accelerometer-based sensor technologies. Five study sections (A–E) were selected to represent a range of road surface conditions, from newly constructed roads to severely deteriorated surfaces. These sections were chosen based on bicycle traffic volume and prior reports of pavement degradation. The evaluation of road surface roughness was conducted using a smartphone-mounted accelerometer to measure the vertical, lateral, and longitudinal accelerations. The data collected were used to calculate the Bicycle Road Roughness Index (BRI) and Faulting Impact Index (FII), which provide a quantitative measure of road conditions and the impact of surface defects on cyclists. Field surveys, conducted in 2022, identified significant variation in roughness across the study sections, with values of BRI ranging from 0.2 to 0.8. Sections with a BRI greater than 0.5 were considered unsafe for cyclists. The FII showed a clear relationship between bump size and cycling speed, with higher bump sizes and faster cycling speeds leading to significantly increased impact forces on cyclists. These findings highlight the importance of using quantitative metrics to assess bicycle lane conditions and provide actionable data for maintenance planning. The results suggest that the proposed methodology could serve as a reliable tool for the evaluation and management of bicycle lane infrastructure, contributing to the improvement of cycling safety and comfort.

## 1. Introduction

Surface roughness is a critical factor influencing the safety, comfort, and overall quality of road infrastructure [1]. As urban and rural cycling networks continue to expand globally, there is a growing need for practical and reliable methods to evaluate bicycle path conditions. Traditional pavement roughness evaluation approaches—such as the Profile Index (PrI) and International Roughness Index (IRI)—typically rely on vehicle-mounted sensors traveling at standard speeds (e.g., 80 km/h) [2,3]. These methods are not applicable to bicycle paths due to limited vehicular access and the distinct characteristics of non-motorized travel. Moreover, IRI and PrI were originally developed for motor vehicles and fail to adequately reflect the dynamic experience of cyclists on narrow, often less-maintained surfaces [3,4]. Recent research has attempted to adapt these indices by using smartphone accelerometers mounted on vehicles or motorcycles. However, the resulting IRI values vary significantly depending on speed, suspension system, and vehicle type, with motorcycles yielding values up to 1.9 times higher than standard vehicles [5]. While GPS and accelerometer-based measurements from bicycles have demonstrated feasibility, most studies have focused on replicating traditional IRI values rather than creating new evaluation models tailored to bicycle infrastructure. This indicates a need for specialized indices that reflect the unique operational and safety considerations of cyclists.

To address this gap, the present study introduces a new roughness evaluation metric—termed the Bicycle Road Roughness Index (BRI)—specifically designed for bicycle roads. Rather than adapting vehicle-based models, BRI is derived directly from field-collected smartphone sensor data under real cycling conditions. It quantifies vertical irregularities that influence ride comfort and is conceptually distinct from skid resistance, which relates to surface friction and is not within the scope of this study. In addition to assessing general roughness, the study also identifies point-specific hazards and proposes a complementary indicator—the Faulting Impact Index (FII)—to evaluate the severity of abrupt surface discontinuities that may pose direct risks to cyclists [6]. In Republic of Korea, the “Bicycle Use Promotion Act” mandates local governments to establish bicycle infrastructure plans; however, only 23% of municipalities have done so [7]. Existing national guidelines for bicycle facility installation and management offer general descriptors for surface conditions—such as “passable but rough” for Grade C—but lack quantitative thresholds or standardized monitoring practices [8,9]. As a result, it remains difficult to assess whether current infrastructure maintenance efforts sufficiently address user safety and ride quality. Although advanced profiling systems have been used in motorized contexts, their high cost and operational complexity limit their application on bicycle-dedicated facilities [8,9].

Smartphone-based accelerometers offer a promising alternative for evaluating pavement conditions due to their affordability, accessibility, and ease of integration into daily cycling. Studies have demonstrated that smartphone-derived acceleration data can correlate with traditional IRI values, supporting their potential use in road surface monitoring [4,10]. Furthermore, mobile applications that capture three-axis acceleration and GPS data now allow for real-time detection of roughness levels and hazardous features [8]. Building upon these foundations, the present study proposes an integrated framework for evaluating bicycle road surface conditions using smartphone sensor technology. Core innovations include the application of a speed correction factor to improve measurement accuracy under varying cycling speeds and the combined use of objective sensor data and subjective user feedback. By addressing previous limitations—such as inconsistent sampling, battery constraints, and the absence of rider-specific models—this approach provides a scalable, low-cost method for assessing diverse bicycle environments. Ultimately, the proposed methodology aims to support the development of safer, more comfortable, and sustainable bicycle infrastructure through data-driven maintenance and planning strategies.

## 2. Materials and Methods

### 2.1. Study Area

This study selected three bicycle routes along Korea’s Four Major Rivers (Geum River, Bukhan River, and Yeongsan River) as the study areas. The Four Major Rivers refer to Korea’s primary waterways, which have played a pivotal role in a national river restoration project. These bicycle paths have evolved into essential components of the nation’s transportation infrastructure, providing cyclists with the opportunity to enjoy the scenic beauty of river landscapes [11]. Each of the three routes spans approximately 100 km, tracing the riverbanks.

**Figure 1:**
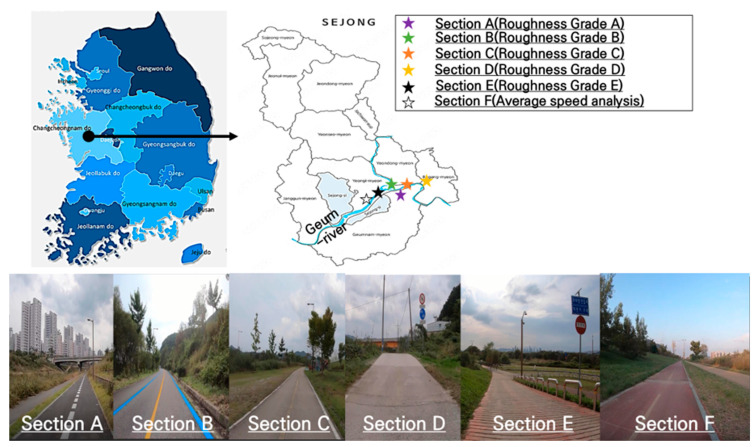
Study area in Republic of Korea to evaluate various bicycle road.

The study sections were chosen based on high bicycle usage and previously documented issues concerning pavement deterioration and maintenance. A preliminary field survey was conducted across the entire bicycle network in Sejong City, along the Geum River, utilizing both walking and cycling to evaluate pavement conditions, materials, and the occurrence of cracks and surface irregularities. The descriptions of the survey sections are depicted in Figure 1. To ensure the comprehensive inclusion of varying service levels described in Table 1, the selected sections represented a spectrum of pavement conditions, ranging from well maintained (A) to those in need of substantial repairs (E). The primary goal of this selection was to obtain reliable reference data to validate the sensor-based pavement roughness measurements. The selection criteria were based on pavement roughness standards outlined in Korea’s Guidelines for the Installation and Management of Bicycle Facilities [12]. Additionally, information on pavement material types, surrounding environments, and usage levels was gathered to facilitate a comparative analysis of spatial patterns in pavement deterioration and hazard distribution. Based on these surveys, five final study sections (A–E) were chosen. For each section, detailed investigations were conducted, including bicycle usage surveys, pavement roughness measurements, and the identification of hazardous elements [8,12].

The field survey of road roughness was conducted in 2022. Section A is an asphalt-paved road, constructed within the year preceding the survey, exhibiting no cracks or resurfacing, and fully meeting the Grade A pavement roughness criteria as outlined in the current guidelines. This section, designated for future urban development, experiences relatively low bicycle traffic. Section B, completed in 2011, is part of the Four Major Rivers bicycle project. This segregated bicycle-pedestrian path is characterized by high bicycle traffic. Section C, a concrete-paved section, has been maintained without significant repairs since 2011. Despite some spalling at certain joints, it remains passable for cyclists, although comfort is reduced. Section D is a concrete-paved road shared by bicycles and vehicles, frequently used by agricultural vehicles and machinery. This leads to common occurrences of spalling, raveling, and cracking, all of which affect the comfort of bicycle travel, though passage is still possible. Section E, a concrete-paved section, severely hinders bicycle travel. Located at the shared entrance/exit between the bicycle path and a riverside parking lot, it features an anti-slip surface that restricts high-speed cycling. While it is a sloped access road rather than a primary bicycle path, it was included to gather data on severely deteriorated pavement conditions. In addition to the roughness assessment, the optimal cycling speed for measuring roughness was determined in Section F, the most congested area near the surveyed sections. However, the hazard analysis was conducted across all sections, focusing on the experimental methodology implementation rather than specific area designation.

### 2.2. Measurement Equipment and Speed Determination for Roughness Evaluation

Measurements were conducted by mounting a Samsung Galaxy S22 smartphone (Samsung, Suwon, Republic of Korea) vertically at the center of the bicycle handlebar, maintaining a uniform speed of 15 km/h to minimize speed-related data deviations. The study utilized a Samsung Galaxy S22 smartphone (Android based, with a built-in 3-axis accelerometer and GPS), the Physics Toolbox Sensor Suite application (Android app) capable of simultaneous 3-axis acceleration and GPS measurement, a handlebar-mounted smartphone holder, an experimental step reproduction device, a distance measuring wheel, and a digital tape measure. All equipment underwent pre-calibration and repeated measurements to ensure data reliability. The experiments were carried out on the same surface condition (concrete pavement) to minimize external influences. Considering sensor sensitivity variations across different smartphone manufacturers, the Samsung Galaxy smartphone, which held the largest domestic market share (76% in Q2 2022), was chosen for the study [13].

For pavement condition measurements, both 3-axis acceleration and GPS data were collected, with data acquisition intervals set to 0.1 s for roughness surveys and 0.01 s (100 Hz) for hazard surveys. The accelerometer recorded acceleration values in the X (lateral), Y (longitudinal), and Z (vertical) directions at a frequency of 10 Hz (0.1 s intervals), with the data stored in Excel file format. The sum of the X-axis, Y-axis, and Z-axis vectors was measured by placing five 3 cm steps adjacent to each other during the data collection period at both 0.1 s and 0.01 s intervals, with each step being impacted 50 times at an approach speed of 25 km/h. A 0.1 s measurement interval corresponds to a distance of approximately 70 cm when traveling at 25 km/h. Consequently, in some instances, the maximum impact generated during step impacts could not be detected. Although five steps of the same size were impacted, the maximum impact value was not detectable, as indicated by the varying scales in the graph. When the same steps were measured at the 0.01 s data collection interval, it was observed that the standard deviation of the impact amount for each step was smaller than at the 0.1 s interval, and the average value also increased. This suggests that a smaller time interval results in more accurate step impact measurements. To evaluate the general pavement condition, the roughness survey was conducted across the entire length of the bicycle road section. using 0.1 s intervals to strike a balance between data volume and battery consumption. In contrast, the hazard survey was performed at 0.01 s intervals to capture rapid 3-axis acceleration changes caused by abrupt pavement irregularities. The choice of data acquisition intervals—0.1 s (10 Hz) for roughness surveys and 0.01 s (100 Hz) for hazard surveys—was determined based on pilot tests and considerations of signal resolution, battery efficiency, and expected surface event frequency. For roughness measurements, a 0.1 s interval was sufficient to capture general undulations over long pavement segments while maintaining data volume and power usage within practical limits. In contrast, hazard detection required high temporal fidelity to capture rapid, high-magnitude impacts caused by short-duration events such as vertical steps or potholes. Pilot experiments showed that lower sampling rates frequently missed peak accelerations during such events, especially at higher cycling speeds. Thus, a 0.01 s interval was applied for the hazard survey to ensure accurate detection and reliable characterization of surface-induced shock events. Previous studies have demonstrated that high-frequency data collection is crucial for accurately capturing rapid surface changes, such as impacts from surface discontinuities [8]. Informed by these findings, this study employed a 0.1 s measurement interval for the roughness survey to efficiently monitor general surface conditions over long distances, while also managing data volume and device battery limitations. For hazard surveys, where capturing short-duration, high-magnitude accelerations is essential, a finer measurement interval of 0.01 s was used to ensure precise detection of sudden surface irregularities. Each measurement point corresponds to a 7 cm distance.

**Figure 2:**
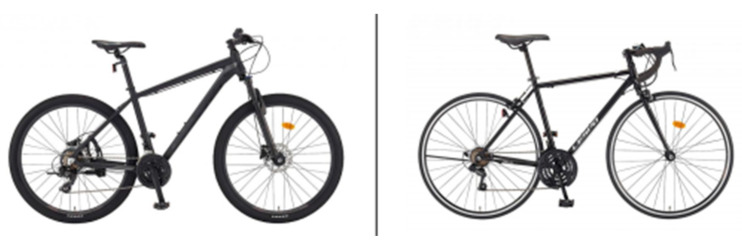
Difference between images of MTB (left) and road bike (right) (Image source: Samchully Bicycle Manufacturing Company website in Republic of Korea, https://www.samchuly.co.kr/index.php/(Accessed on 20 April 2025.).

In this study, a road bicycle equipped with 700 c tires was used as the measurement device for assessing pavement roughness. Mountain bikes (MTBs) with 26-inch wheels were excluded due to their diverse suspension systems and varying tire sizes, which could attenuate surface impacts and affect the accuracy of the measurements. Road bicycles, developed in Europe, use wheel sizes measured in centimeters, while mountain bikes (MTBs), developed in the United States, use wheel sizes measured in inches [14,15]. MTBs include hardtail models (with front suspension only) and full-suspension models (with both front and rear suspensions), which have variables such as suspension travel length (100–200 mm), damping strength, and tire width (2–3 inches) [16]. To minimize these factors and ensure the accurate transmission of surface impacts to the sensor, a road bicycle—known for its greater sensitivity to pavement conditions—was selected for this study. The photo of the bicycle used in the experiment is shown in Figure 2.

Low tire pressure in bicycles can absorb excessive road shocks, negatively impacting the accuracy of sensor measurements. Typically, the maximum tire pressure for road bikes ranges from 90 to 110 psi, with slight variations depending on the wheel and tire type. To ensure optimal measurement sensitivity, the maximum tire pressure indicated on the wheels and tires was maintained throughout the study. This setup was based on a pilot test that showed lower tire pressure significantly reduced the transmission of surface-induced shocks to the handlebar sensor due to increased pneumatic absorption. Maintaining the maximum allowable tire pressure minimized this damping effect and ensured that road surface irregularities were accurately captured by the accelerometer without attenuation. This condition was essential for validating the sensitivity and responsiveness of the sensor system to pavement roughness.

**Figure 3:**
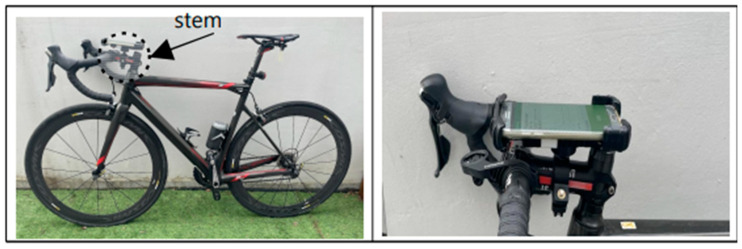
Bicycle and smartphone mounting methods for road surface measurement.

The smartphone mounting position was determined based on related research suggesting that this positioning minimizes the impact of road surface irregularities and vibrations on sensor measurements [17]. In this study, a mounting system without shock absorption was selected to prevent external shocks from affecting sensor readings. Additionally, a system with an anti-detachment locking feature was used to prevent the smartphone from detaching due to strong road impacts. The smartphone was installed parallel to the bicycle’s ground surface shown in Figure 3, with its top aligned with the direction of travel, ensuring that the sensor axes matched the direction of the bicycle’s movement. To ensure that the smartphone remained in a ground-parallel position throughout the experiment, a manual bubble level tool was used before each trial to check the horizontal alignment of the smartphone mount. The mount was adjusted until the level indicator was centered, confirming parallel placement relative to the ground. Additionally, a rigid, non-damping mounting bracket with an anti-rotation locking mechanism was employed to prevent any angular displacement during measurement. The orientation of the smartphone axes was verified using the Physics Toolbox Sensor Suite application to confirm that the x-, y-, and z-axes were aligned with lateral, longitudinal, and vertical motion, respectively. Measurements were repeated for each section, and any instance where displacement or misalignment was observed post-survey was discarded and re-measured to maintain data accuracy and reliability.

This alignment prevented data distortion, thereby enhancing the accuracy and reliability of the measurements.

**Figure 4:**
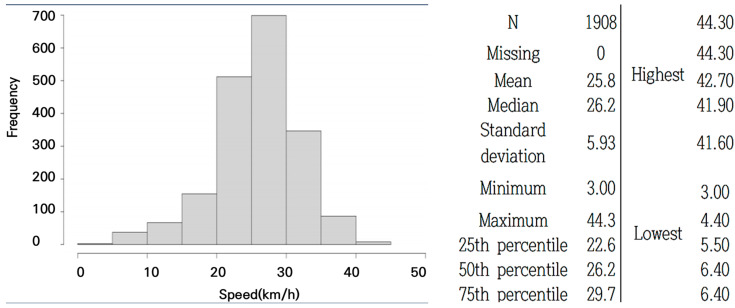
Distribution of bicycle riders’ travel speeds using the Strava App (www.strava.com, accessed on 20 October 2022.).

The riding speed for measuring road roughness was determined based on typical cycling speeds. A 3 km stretch in Section F, with a 0.0% gradient (flat road), was selected to identify the optimal speed for measuring bicycle road roughness. From January to November 2022, the riding speeds of 1908 cyclists using the Strava app in this section were recorded. The average riding speed was found to be 25.8 km/h, and a standard riding speed of 25 km/h was selected for the road roughness measurements. The statistical results are presented in Figure 4.

A correction factor for speed variations was introduced to allow bicycle path managers to adjust measurements according to local riding patterns. Since Strava is the most widely used bicycle tracking app, data analysis was initially conducted using this app to validate the appropriateness of the speed measurement. Based on the collected speed data, the bicycle path was categorized into three speed groups—15 km/h, 20 km/h, and 25 km/h—for measuring travel speed in hazardous sections. The impact of speed variations on bump-induced impacts was then assessed for each speed category.

### 2.3. BRI Calculation

The smartphone’s 3-axis accelerometer measures acceleration changes along the x, y, and z axes. When the smartphone is positioned parallel to the ground, the x-axis corresponds to the left-right direction, the y-axis corresponds to the forward-backward direction, and the z-axis corresponds to the vertical direction [6,8]. Since measurement data can vary depending on the smartphone’s orientation, all surveys were conducted with the smartphone mounted on the bicycle stem, parallel to the ground, and with the top of the smartphone facing the direction of travel. Additionally, to accurately measure riding speed, a bicycle speedometer was installed. Unlike GPS-based speedometers, wheel rotation-based devices minimize signal errors; hence, this approach was adopted in our study. For road roughness measurement, the acceleration change along the vertical z-axis was primarily monitored to reduce the influence of handlebar steering by the cyclist. The measured data were then averaged using the absolute values for the section. The primary BRI was defined as the average of the absolute vertical acceleration changes during the travel section, with additional refinement provided by the following equation, as determined through field surveys. Although the BRI is based on a simplified representation using vertical acceleration, it effectively serves as a practical proxy for dynamic ride quality, reflecting the level of pavement-induced vibration perceived by cyclists in real conditions.

This equation is shown in Equation (1).

(1) $$BRI=\frac{\sum \left(\left|\underset{Z_{1}}{\to }\right|+\left|\underset{Z_{2}}{\to }\right|+\ldots +\left|\underset{Z_{n-1}}{\to }\right|+\left|\underset{Z_{n}}{\to }\right|\right)}{T}\;=\frac{(\sum (\left|\underset{Z_{1}}{\to }\right|+\left|\underset{Z_{2}}{\to }\right|+\ldots +\left|\underset{Z_{n-1}}{\to }\right|+\left|\underset{Z_{n}}{\to }\right|))*V}{T}$$

T$:driving\;speed\left(s\right)=sampling\;interval\;is\;per\;0.01\;s$
L:driving distance
V$:driving\;speed(\frac{km}{h})$

This index quantitatively measures the vertical impact experienced by bicycle riders during travel. A higher value indicates greater road roughness deterioration and lower riding comfort. In this study, the BRI was used to compare and analyze the road roughness levels across various sections of the surveyed areas.

### 2.4. Acceleration Sensor Measurements for Risk Point Determination

**Figure 5:**
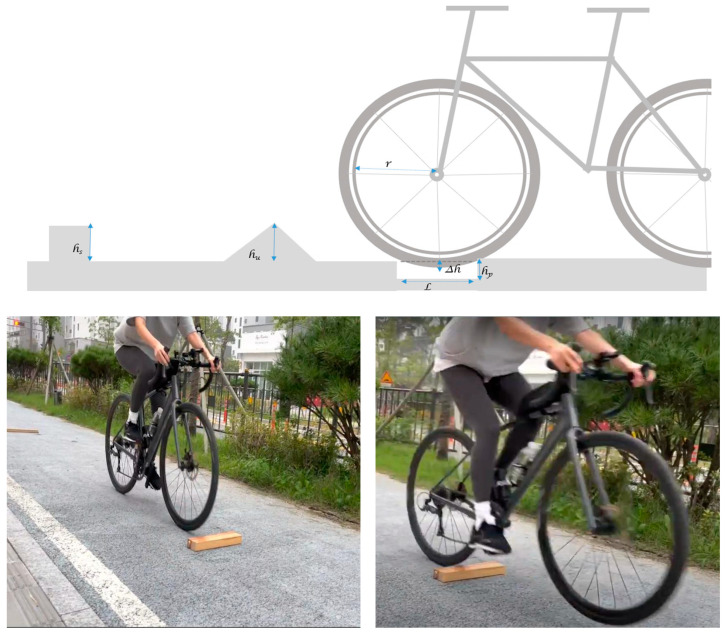
Concept diagram and simulation experiment to find acceleration changes at hazardous points using bicycle.

Risk assessment was performed to identify points on the bicycle path that pose a risk of accidents and to quantify the level of risk. Risk factors were categorized into potholes, step differences, sinkholes, faulting, and others. Unlike roughness factors, which are distributed over longer stretches, risk factors tend to appear in short segments or specific points, making them more likely to cause sudden impacts that could lead to accidents. To classify these risk factors, upward and downward step differences were analyzed, along with other potential hazards on the bicycle path. Experiments were designed to measure the impact of these risk factors by varying their size and the riding speed presented in the concept of Figure 5. While various types of risk factors exist on road pavements, they can be categorized based on the type of impact they have on bicycles. Specifically, risk factors are divided into upward and downward step differences, with potholes and sinkholes—despite originating from different causes—being grouped with road heaving and other protrusions as upward step differences.

In Equation (2), when the diameter of a pothole (L) is smaller than the size of the bicycle wheel (2r), the wheel does not reach the bottom of the hole, causing only a small drop and resulting in a smaller acceleration change compared to the step difference. Therefore, in this study, various risk factors occurring on bicycle path pavements were classified into upward and downward step differences, and the changes in 3-axis acceleration based on the size of these step differences were measured.

(2) $$∆h=r-\sqrt{r^{2\;}-{\left(\frac{L}{2}\right)}^{2}<h_{p}}\;\left(That.\;L\leq 2r\right)$$

$h_{p}=∆h\;($ That. L > 2r)
∆h = Maximum height of pothole step differenc*e*
r = wheel radiu*s*
L = pothole diameter
$h_{p}\;=\;pothole(\;or\;sinkhole\;depth,$ downward step differences)

When the depth (or height) of the hazard is the same, the impact force of the step difference is greater than that of the pothole. This relationship is described in Equation (3).

(3) $$∆{ah}_{s}\geq ∆{ah}_{p}\geq ∆{ah}_{M}$$

$∆{ah}_{s}=3-axis\;linear\;acceleration\;change\;of\;the\;step\;(m/s²$)
$∆{ah}_{p}=$3-axis linear acceleration change of the pothole (m/s²)
$∆{ah}_{M}=3-axis\;linear\;acceleration\;change\;of\;the\;road\;surface\;bulge\;(m/s²)$

In this study, which aims to detect risk factors, the impact index generated during a collision was measured based on the step, the most sensitive factor in the event of a bicycle collision. Upward and downward step experiments were designed, with a preliminary experiment adjusting the height by fixing wooden square timber for the upward step and wooden plates for the downward step to the ground. The test used 3 cm high wooden square timber. For the upward step difference, wooden square timbers were fixed to the road surface to adjust the height, while the downward step difference experiment involved fixing wooden panels to the ground and adjusting the height to conduct preliminary tests. These experiments measured the acceleration changes caused by different step sizes to evaluate potential risk factors when a bicycle collides with these obstacles.

### 2.5. Risk Assessment Survey Method Using Step Difference Experiment

Using the results of the acceleration sensor measurements for risk point determination, a field survey is conducted to assess the risk on bicycle roads. To identify hazard points, the sum of the 3-axis vector magnitudes (SVM, signal-vector magnitude) is utilized. The concept of SVM is an algorithm for classifying static and dynamic posture changes, implemented using the triaxial acceleration information measured by the current system [18]. In this study, this value is referred to as the Faulting Impact Index (FII), which quantifies the impact caused by step differences. This approach provides a more accurate measurement of the effects of upward and downward steps on bicycle handling and offers a clear criterion for classifying hazard elements. The calculation equation is shown in Equation (4).

(4) $$FII\left({SVM}_{I}\right)=\sqrt{{∆x_{i}}^{2}+{∆y_{i}}^{2}+{∆z_{i}}^{2}}$$

$FII\left({SVM}_{I}\right)\;=\;Step\;impact\;index,Sum\;of\;themagnitudes\;of\;the\;3-axis\;acceleration\;vector$*s*
${∆x_{i}}^{2}\;=\;Change\;in\;x-axis\;acceleration\;at\;point$ *i*
${∆y_{i}}^{2}\;=\;Change\;in\;y-axis\;acceleration\;at\;point$ *i*
${∆z_{i}}^{2}\;=\;Change\;in\;z-axis\;acceleration\;at\;point$ *i*

In the experimental setup for measuring hazard levels, upward steps were created by fixing wooden square timber to the ground, while downward steps were formed by stacking wooden panels to match the height of curbstones, with step heights adjusted by removing panels. These experiments were designed to replicate real-world hazards encountered by cyclists on bicycle paths, specifically focusing on step differences (both upward and downward), as well as other potential hazards such as potholes and sinkholes. The aim was to assess the impact of various step heights (1 cm, 2 cm, 3 cm, and 4 cm) and cycling speeds (15 km/h, 20 km/h, and 25 km/h) on the acceleration changes experienced by cyclists. By doing so, the study analyzed how different step sizes and speeds contribute to the risk of accidents, particularly by measuring the intensity of impacts during encounters with these hazards. The primary objective was to establish a coefficient for hazard severity and to understand how these factors influence rider safety, thereby providing valuable data for hazard classification and maintenance planning on bicycle paths. The experimental setup method for measuring hazards on bicycle roads is described in Table 2.

### 2.6. Experiment on Faulting FII Based on Step Size and Speed Variations

The experiment was conducted to calculate the FII by measuring the acceleration changes experienced by the bicycle under different step sizes and riding speeds. A total of nine scenarios were designed, combining three cycling speeds (15 km/h, 20 km/h, and 25 km/h) with three step heights (10 mm, 20 mm, and 30 mm). Each scenario was repeated 50 times to observe variations in acceleration across the x, y, and z axes. For each impact event, the sum of the maximum acceleration values from all three axes, referred to as the impact index, was recorded. To minimize GPS-related errors and ensure accurate speed control, a Garmin Edge device—capable of measuring wheel rotations—was utilized. The Garmin Edge is a GPS-enabled bicycle computer that supports navigation, performance tracking, and data analysis features for cyclists. All experiments were carried out in a spacious environment to maintain consistent speeds throughout the tests. The road bicycle used in the experiment was equipped with 700 c wheels and maintained a maximum tire pressure of 110 psi. Acceleration data were recorded by smartphone sensors at 0.01 s intervals. In the experimental setup, wooden square timbers were firmly fixed to the ground using drills and screws to prevent any displacement during impact. For downward step simulations, wooden panels were stacked to match the height of curbstones, and the step height was adjusted by removing layers of panels as needed. The detailed configuration of the experimental design is summarized in Table 3.

## 3. Results

### 3.1. Results of BRI with the Different Bicycle Road Roughness Grade

**Figure 6:**
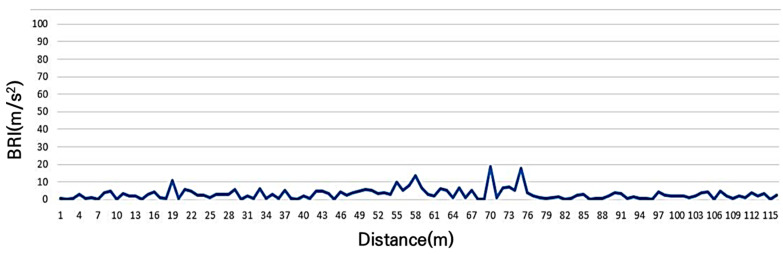
Bicycle road roughness index (BRI) of grade A (high level of ride comfort).

Using a road bike with a tire pressure of 110 psi, the BRI was measured at each point while riding at a speed of 20 km/h across arbitrarily selected expected service levels ranging from A to E. As shown in Figure 6, the estimated BRI for Section A, which represents a newly constructed bicycle road anticipated to have excellent surface roughness, was 3.0 m/s^2^. The maximum recorded value was 18.7 m/s^2^, the minimum value was 0.0 m/s^2^, and the standard deviation was 3.1 m/s^2^, indicating favorable pavement conditions.

**Figure 7:**
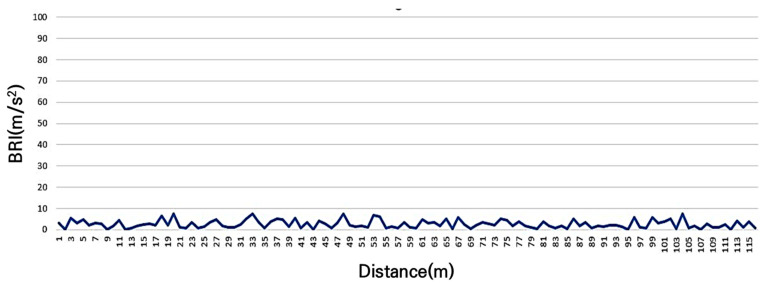
BRI of grade B (not smooth as newly constructed but excellent roughness).

The BRI for Section B, shown in Figure 7, which has been well maintained for at least 11 years since construction, was 2.2 m/s^2^. The maximum recorded value was 7.5 m/s^2^, the minimum value was 0.0 m/s^2^, and the standard deviation was 1.9 m/s^2^. Despite the passage of 11 years, the pavement quality at the time of construction was excellent, resulting in smoother roughness compared to the newly constructed Section A.

**Figure 8:**
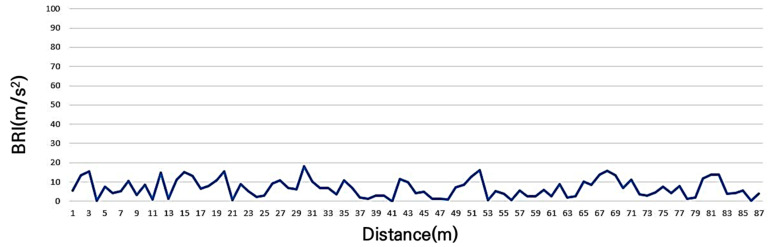
BRI of grade C (some roughness degradation such as surface labeling).

The 11-year-old concrete-paved bicycle road on the south side of the river, which was expected to correspond to service level C, showed a BRI of 6.8 m/s^2^. The maximum value was 17.9 m/s^2^, the minimum value was 0.05 m/s^2^, as shown in Figure 8. And the standard deviation was 4.7 m/s^2^, indicating that partial pavement damage is occurring on the surface.

**Figure 9:**
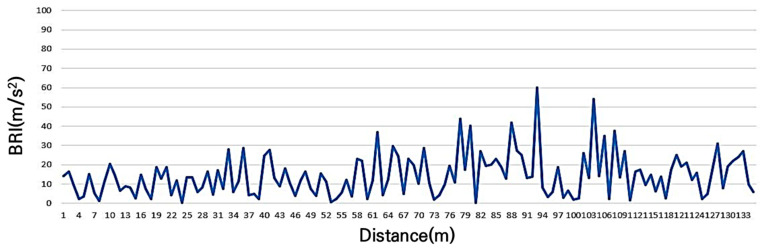
BRI of grade D (significant surface deterioration like labeling, cracking).

The 11-year concrete-paved shared bicycle and automobile road along the riverside, where spalling and cracks were visually observed and a service level D was expected, exhibited a BRI of 14.5 m/s^2^, as shown in Figure 9. The maximum recorded value was 60.0 m/s^2^, the minimum value was 0.24 m/s^2^, and the standard deviation was 10.9 m/s^2^, indicating that the BRI measured using the smartphone sensor accurately reflects the actual surface condition.

**Figure 10:**
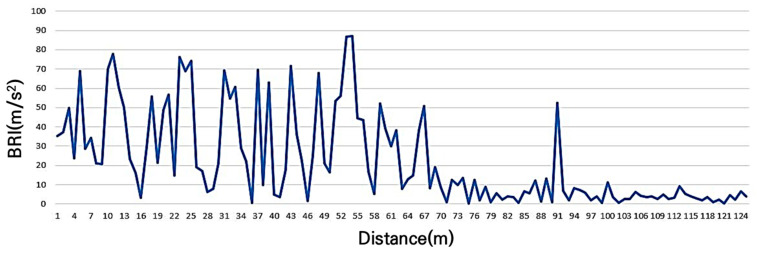
BRI of grade E (simulate driving difficulty due to excessive non-slip).

Although not part of the main bicycle path, this section serves as an access road to both the bicycle path and a parking lot. Due to the anti-skid pavement, significant vibrations are felt on the surface, making it difficult to use. Service level E was expected, and the BRI was 22.6 m/s^2^. The maximum recorded value was 87.0 m/s^2^, the minimum value was 0.21 m/s^2^, and the standard deviation was 24.0 m/s^2^, indicating that the BRI measured by the smartphone sensor accurately reflects the perceived surface condition. This is presented in Figure 10.

The IRI for the expected service levels A to E was measured and reviewed. As described in Table 4, most of the measurements aligned with the predicted levels. However, in the newly constructed route, Scenario 1, the surface roughness was worse than Scenario 2, which had been well maintained. Both Scenario 1 and Scenario 2 are asphalt roads, with a 10-year difference in their construction years. Despite this, the surface roughness in Scenario 2, which was excellently paved during the initial construction of the bicycle lane, was found to be better. This finding can be attributed to the long-term effects of traffic load and environmental factors such as weather, which significantly affect the pavement’s performance over time. While new pavements generally exhibit lower roughness, the quality of the initial construction, maintenance practices, and traffic volume over time are more influential in determining the long-term pavement roughness [19]. The better surface condition of Scenario 2 suggests that excellent initial construction and regular maintenance practices contribute to more sustainable pavement quality, which may help maintain smoother surfaces even after years of use. Moreover, the findings support the notion that surface roughness in bicycle lanes is less affected by lighter bicycle traffic compared to roads designed for heavier vehicle use [20]. Thus, the quality of initial construction and maintenance becomes crucial in determining the durability and rideability of bicycle lanes.

### 3.2. Improvement of BRI Model for Minimizing Speed Error

Through field surveys conducted to gather data on road surface roughness, a notable disparity in the BRI values was observed between areas characterized by smooth surfaces and those exhibiting significant roughness. This finding confirmed the viability of utilizing smartphones for surface roughness surveys. However, it was anticipated that maintaining a consistent speed of 25 km/h without error would prove challenging due to human factors, especially as the length of the survey area was extended. In an effort to refine the existing BRI model (Equation (1)), experiments were designed to observe changes in surface roughness in response to varying bicycle speeds presented in Table 5. The results from these experiments were subsequently integrated into the model. Given that bicycle speed directly influences sensor readings, this study focused on calculating BRI correction factors specific to speed.

**Figure 11:**
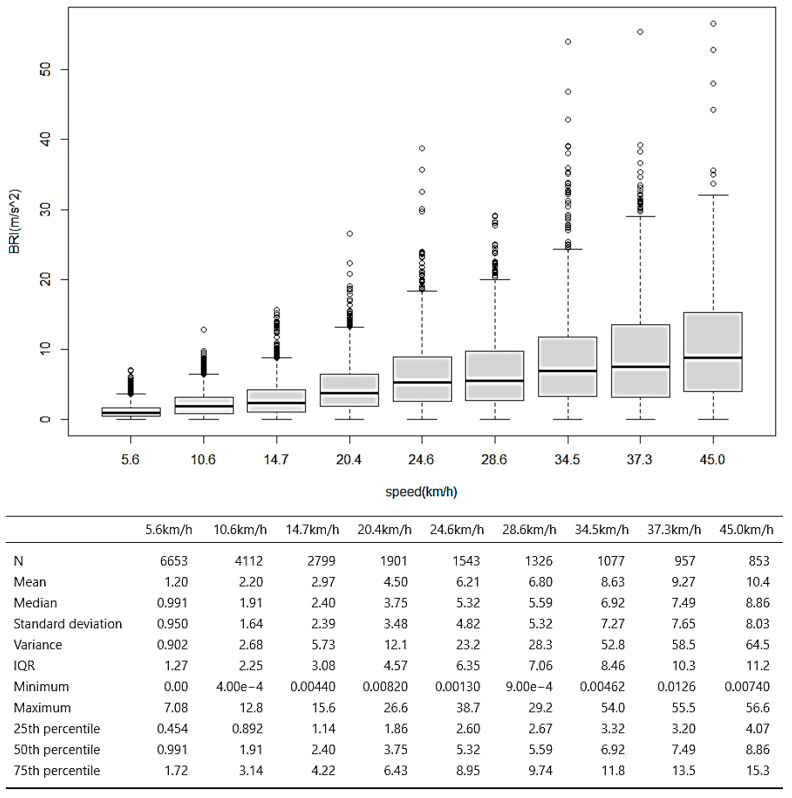
Statistical analysis results of BRI changes according to riding speed.

The experiment involved traversing the same section at incrementally increasing speeds, ranging from 5 km/h to 45 km/h in 5 km/h intervals. A distance of 150 m was covered, with the experiment repeated 30 times to ensure reliability. During each trial, the cyclist aimed to maintain the target speed, while actual speed was calculated using GPS data from the smartphone. Human factors introduced discrepancies between the intended and actual cycling speeds. When measuring personal mobility speed, such as bicycles, errors can occur due to human factors. In particular, errors in the measured speed may occur due to differences in the speed control method depending on the driving environment, the user’s experience level, and safety consciousness [21]. Consequently, the analysis of BRI values was based on the actual measured speed rather than the target speed. Detailed findings and further statistical analysis are presented in Figure 11.

The analysis of GPS-measured speed and corresponding BRI values revealed that, while the road section maintained consistent roughness, an increase in bicycle speed led to more pronounced changes in surface roughness, as measured by the z-axis acceleration of the smartphone sensor.

This suggests that higher speeds enhance the sensor’s sensitivity to surface irregularities, indicating that as the speed of the bicycle increases, the variation in the sensor’s readings becomes more significant due to the dynamic response of the bike to surface roughness. Consequently, this underscores the necessity for speed-adjusted corrections to ensure accurate BRI measurements across varying conditions. Such corrections are essential for refining the model and enhancing the reliability of surface roughness assessments in real-world cycling environments.

**Figure 12:**
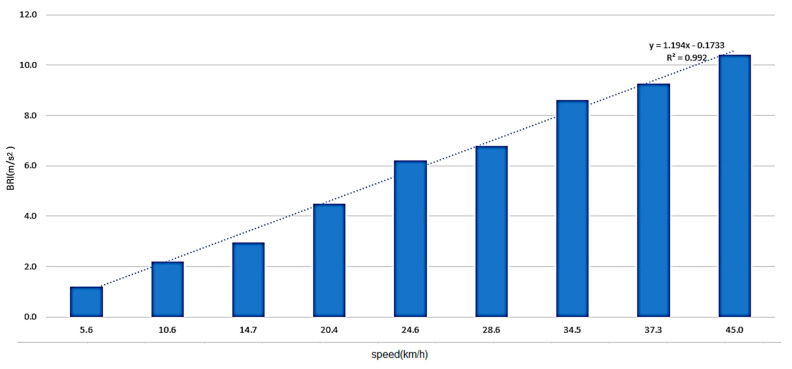
Results of surface roughness changes based on bicycle speed variations.

Therefore, the results of surface roughness changes based on bicycle speed variations, as shown in Figure 12, can be explained as follows: When traversing the same section, the variation in surface roughness with increasing speed can be modeled using a linear regression equation. The surface roughness BRI (dv), corresponding to the average speed of the section, can be represented as shown in Equation (5).

(5) $$\mathrm{BRI}(\mathrm{dv})=1.194\times \bar{v}-0.1733$$

BRI:Bicycle Road Roughness Inde*x*
$\bar{v}:Average\;bicycle\;spee$*d*

BRI measurements are typically conducted at a speed of 25 km/h. However, due to both human and environmental factors, maintaining a consistent speed of 25 km/h proves challenging. To address this issue, Equation (1) was modified to Equation (6) to account for the section’s average speed. The speed correction factor is applied based on the average speed for the minimum measurement unit, which is recommended to be 100 m. Additionally, outliers are excluded when the actual bicycle speed, as depicted in Figure 3, falls below 6 km/h or exceeds 42 km/h.

(6) $$B\mathrm{RI}=\frac{(\sum_{i}^{j}\left|\underset{Z_{i}}{\to }\right|)}{t_{ij}}\text{×}{s\mathrm{cf}}_{\mathrm{ij}}\;(\mathrm{if},\;6\;\mathrm{km}/h\;\text{≤}{\;v}_{i}\;\text{≤}\;42\;\mathrm{km}/h)$$

BRI:Bicycle Road Roughness Inde*x*
$Z_{i}:Vertical\;acceleration\;variation\;at\;point$ *i*
*j Time to traverse the unit section for roughness evaluation* (*Recommended section length*: 100 *m*)
$t_{i}:Traversal\;time\;of\;i-jsection\;(Unit\;:0.1sec$
${scf}_{ij}:Speed\;Correction\;Factor\;of\;i-j\;sectio$*n*
$v_{i}:Speed\;at\;point$ *i*
$\bar{v_{ij}}:Average\;speed\;of\;i-j\;sectio$*n*

The correction coefficient based on the BRI model, adjusted for the average speed in Section F using the apps, was presented for speeds ranging from 6 km/h to 42 km/h, referring to the driving distribution of the bicycle road. It is recommended that surface roughness measurements be conducted within a typical speed range of 20 km/h to 30 km/h. In this study, the speed correction factors were derived from experimental data collected at 25 km/h, as outlined in Table 6. To better understand specific surface roughness issues on bicycle paths, the survey was conducted based on the 100 m section suggested in this study, and the calibration coefficient corresponding to the average speed within this 100 m section was applied. As shown in the previously presented results, the higher the speed, the lower the calibration coefficient of BRI, which could be presented within the range of 0.59 to 4.25. It is expected that the flatness of each section of bicycle paths can be more accurately assessed, and the errors in the measurement of each section can be minimized by utilizing the results of these studies.

### 3.3. Three-Axis Acceleration Changes When Measuring Flatness and Risk

**Figure 13:**
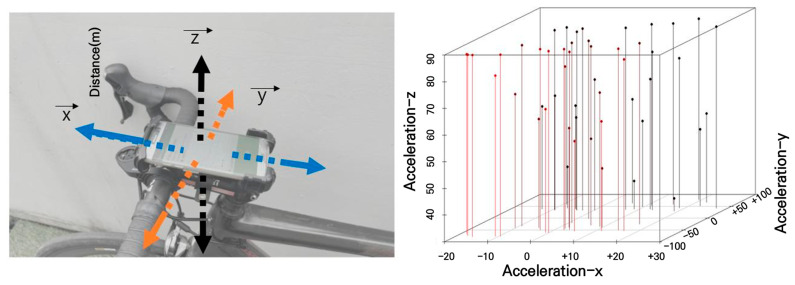
Changes in three-axis acceleration due to bicycle riding(Red line Acceleration-x and black line Acceleration-y refers to the acceleration points and locations with smartphone sensors measurement).

The purpose of this study is to detect risk factors by measuring the impact index that occurs during a bicycle collision, with a specific focus on step differences, which are the most sensitive factor in terms of acceleration changes. In fact, the change in the three-axis acceleration was found to be diverse during the bicycle’s driving process. The acceleration measurements used to determine the risk points indicated that the bicycle handle steering, influenced by the step, was significantly affected, as shown in Figure 13. Therefore, it is crucial to minimize errors caused by human factors through a detailed analysis of these sections in order to accurately determine the risk points.

**Figure 14:**
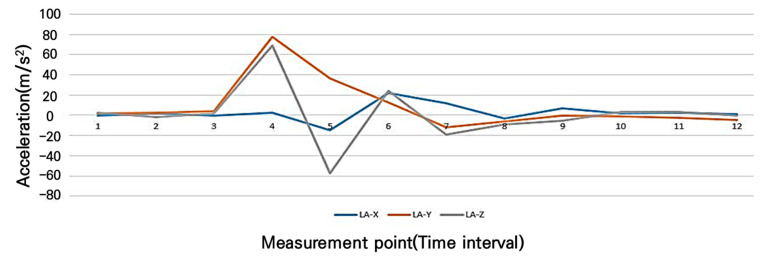
Three-axis acceleration changes with driving step difference impact.

To identify hazard points, acceleration sensor measurements were conducted, as illustrated in Figure 14. The impact from an upward step difference on the road affected the bicycle’s handlebar steering. The results revealed significant changes in acceleration along the direction of travel (y-axis), as well as in the lateral (x-axis) and vertical (z-axis) directions. The results confirm that surface impacts generate significant disturbances across all three axes, affecting rider stability, confirming that an upward step difference on the road can significantly influence bicycle handling.

### 3.4. FII Based on Surface Irregularities

**Figure 15:**
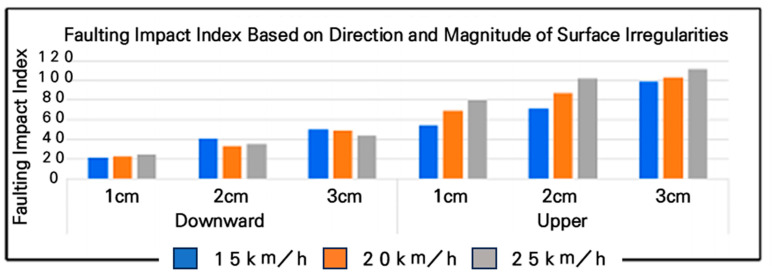
Faulting Impact Index (FII) based on direction and magnitude of irregularities.

The results revealed significant differences in the FII experienced by cyclists, depending on the direction of the step. In experiments conducted at various speeds (15–25 km/h) with five repetitions per step, upward steps produced an FII approximately 2.5 times greater than that of downward steps. Despite the constant step height, the direction of the cyclist’s approach determined whether the step was categorized as upward or downward. Therefore, future experiments focused on measuring FII will prioritize upward steps, which induce greater impact, and exclude the less impactful downward steps. The results of FII based on the direction and magnitude of irregularities on bicycle roads are described in Figure 15. Furthermore, experiments with a step height of 4 cm revealed hazardous situations, such as tire punctures, due to the severity of the impact. As a result, steps exceeding 4 cm in height were classified as highly hazardous. Consequently, this study suggests that the ideal FII range should be between 1 and 3 cm for safe bicycle road conditions.

**Figure 16:**
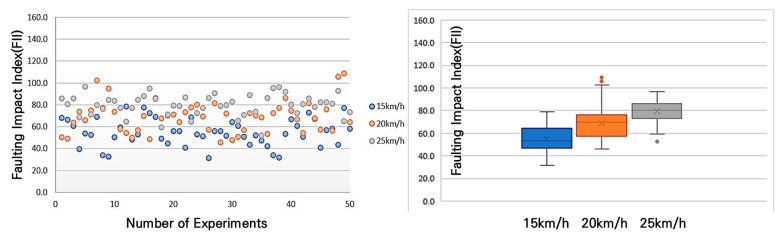
FII by riding speed for a 10 mm bump height.

In accordance with Figure 16, the results of the average shock index measurement for different bump heights and speeds showed that at a 10 mm bump, the shock index was 55 m/s^2^ at a speed of 15 km/h, 69 m/s^2^ at 20 km/h, and an average of 80 m/s^2^ at 25 km/h. This trend—higher FII values at increased speeds—is consistent with established bicycle dynamics; however, in this study, the result serves as a practical validation of the sensor system’s ability to capture speed-sensitive impact data. This confirmation was necessary to ensure that the accelerometer could reliably detect variations in pavement-induced shocks under realistic riding conditions and to define the operational speed range for field measurements.

**Figure 17:**
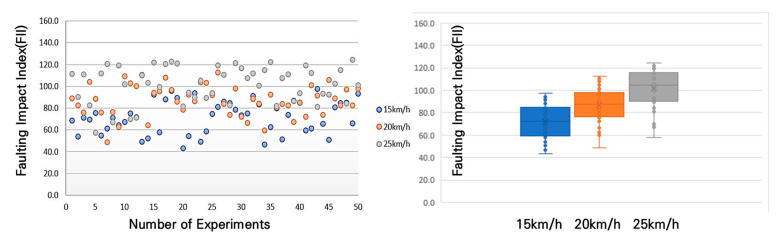
FII by riding speed for a 20 mm bump height.

An analysis of Figure 17 reveals that for the 20 mm bump, the average shock index was 72 m/s^2^ at 15 km/h, 87 m/s^2^ at 20 km/h, and 102 m/s^2^ at 25 km/h.

**Figure 18:**
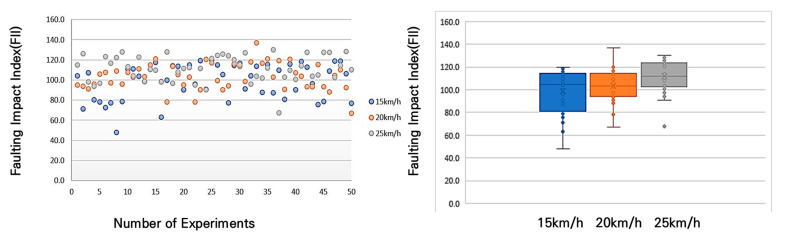
FII by riding different speed for a 30 mm bump height.

Referring to Figure 18, it can be observed that for the 30 mm bump, the average shock index was 99 m/s^2^ at 15 km/h, 103 m/s^2^ at 20 km/h, and 111 m/s^2^ at 25 km/h. These results indicate that, as the bump height increases, the shock index also increases, with higher speeds further amplifying the impact experienced by the cyclist.

**Figure 19:**
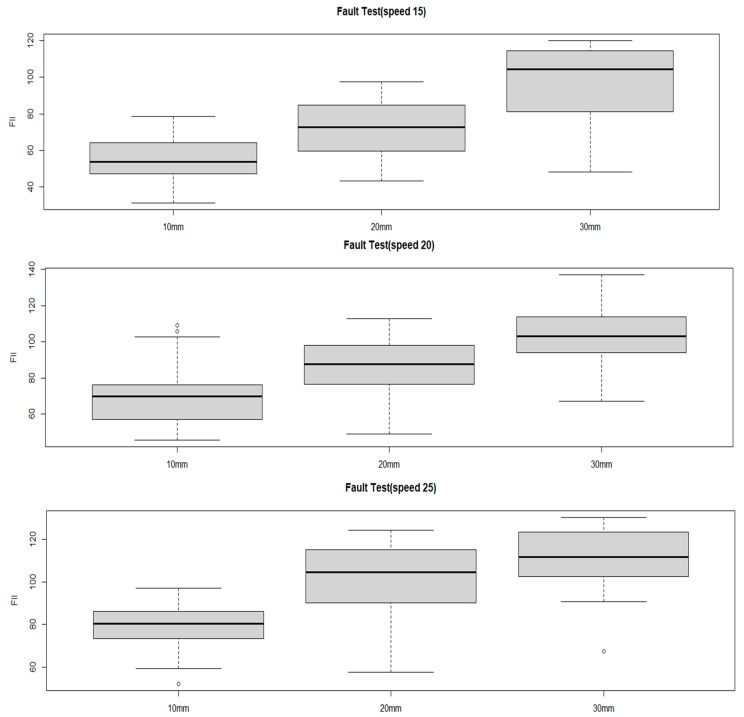
FII results by riding speed for a 10mm, 20mm, 30 mm bump height.

Examining the distribution of the FII by bump size at different driving speeds, at 15 km/h, the FII increased by approximately 130% when the bump size increased from 10 mm to 20 mm and by approximately 137% when it increased from 20 mm to 30 mm. In the 20 km/h experiment, the FII rose by approximately 126% when the bump size increased from 10 mm to 20 mm and by approximately 119% when it increased from 20 mm to 30 mm. In the 25 km/h experiment, the FII increased by approximately 127% when the bump size increased from 10 mm to 20 mm and by approximately 109% when it increased from 20 mm to 30 mm. These findings are presented in Figure 19, with a detailed statistical analysis provided in Table 7. This analysis highlights that as bump size increases, the FII rises significantly, and the rate of increase becomes more pronounced at lower speeds, indicating that smaller bumps may have a disproportionate impact at lower cycling speeds.

Table 7 presents the detailed statistics of the FII across different driving speeds and bump conditions. Overall, the average FII increased with both greater bump heights and higher speeds. At 15 km/h, the average FII ranged from 54.8 m/s^2^ to 98.8 m/s^2^, whereas at 25 km/h, it ranged from 79.9 m/s^2^ to 116.6 m/s^2^, indicating a consistent upward trend. The median values closely aligned with the averages, suggesting a relatively symmetrical distribution of measurements across scenarios. Additionally, the standard deviation values, which ranged from 9.6 to 17.6, indicate moderate variability influenced by both driving speed and bump size. Particularly in scenarios involving higher speeds and larger bumps (e.g., scenarios S6 and S9), both the maximum and minimum FII values increased significantly, highlighting the intensified severity of impacts experienced by cyclists. These findings emphasize the critical need for managing surface discontinuities, especially on paths intended for higher-speed bicycle travel.

As a result of the experiments, the impact index generally increased proportionally with both the step size and bicycle speed. The average FII based on the driving speed and the size of the step difference is shown in Table 8. However, it is presumed that in certain sections, riders unconsciously prepared for shocks by adjusting their arms and legs for absorption, although this study did not account for such human factors. To minimize these influences, changes in impact acceleration were measured on bicycle paths, and user satisfaction with surface conditions was evaluated through a graded survey [22]. It is suggested that panel analysis involving actual cyclists be conducted to further reduce human variability and support interpretation in sections where direct measurement is challenging. Nonetheless, the observed trend—that the impact index increases with both step size and speed—aligns with previous findings showing that the impact acceleration transmitted to bicycles increases with bicycle speed and the magnitude of step differences [8,23].

While the BRI values presented here were used to classify pavement service levels based on field observations, future studies may benefit from correlating BRI with established metrics such as the International Roughness Index (IRI) to further validate and refine evaluation thresholds.

## 4. Discussion

This study examined the current state of bicycle paths to address road roughness issues and proposed an innovative method to help road maintenance managers efficiently identify problem areas. The findings confirmed that although the overall roughness of a bicycle path may be acceptable, localized step differences at specific points can present significant safety hazards to cyclists. Consequently, this study developed a method to measure both general roughness and critical hazardous points separately, overcoming the limitations of conventional road roughness measurement equipment, which often cannot assess dedicated bicycle paths. The method leverages smartphones equipped with 3-axis accelerometers and GPS sensors to evaluate road conditions during cycling. While the speed–impact relationship is well known, its inclusion in this study served a specific methodological function: to verify that the shock magnitude recorded by the smartphone sensor responds appropriately to both speed and surface irregularity, thereby validating the use of FII as a field-applicable metric.

This approach shows promise in measuring and assessing the pavement condition of bicycle paths using only a smartphone and a bicycle, without the need for specialized survey equipment. This makes the method a cost-effective and accessible solution for bicycle path maintenance. While the proposed method shows strong potential, several limitations must be acknowledged.

First, human factors may introduce measurement errors. Cyclists may unconsciously adjust their posture to absorb shocks, particularly in areas with significant step differences, which could affect the accuracy of the measurements. This was particularly evident when cyclists reached speeds of 25 km/h or higher and when step heights exceeded 30 mm. Future research should account for these human factors by developing methods to correct for such errors, especially as more large-scale data from a broader range of cyclists becomes available.

Second, the scope of data collection is limited by the nature of cyclist movement, which typically follows a linear path. Consequently, the measurement range is confined to a narrow width of approximately 2–3 cm, roughly equivalent to the width of a bicycle tire, on a 1.5 m wide bike lane. To address this limitation, future measurements should involve intentionally cycling over the most vulnerable sections of the road, or data should be collected from a larger and more diverse group of cyclists to fill in data gaps. By gathering comprehensive data across multiple riders and sections, road maintenance managers will be better equipped to identify and efficiently manage vulnerable areas of the bicycle path, ultimately ensuring safer cycling conditions.

There is room to integrate more detailed quantitative analysis of the step difference’s effects on safety, including crash statistics or cyclist injury data related to specific road irregularities. By correlating these findings with the FII values, future studies could provide even stronger evidence for the proposed methodology’s practical application in road maintenance. Additionally, it may be valuable to investigate how different types of road materials interact with the proposed measurement system, as various surface types could affect the sensor data. This would help refine the method for broader applications. Future technological advancements could also be considered, such as incorporating machine learning algorithms to analyze sensor data, which would enhance the detection of hazardous points with greater precision, ultimately improving road maintenance efficiency. While this study relied on controlled data collection using a single standardized measurement setup, future research could explore the use of crowdsourced data from everyday cyclists to expand spatial coverage and long-term monitoring capacity. Once the measurement protocol is sufficiently standardized, leveraging user-collected smartphone data may offer a scalable and cost-effective solution for citywide or national-level bicycle infrastructure management.

## 5. Conclusions

This study aimed to provide an effective, accessible method for evaluating the roughness and safety of bicycle lanes, which are rapidly expanding as part of national infrastructure initiatives aimed at promoting eco-friendly transportation and improving public health. While the national bicycle lane network has significantly grown over the past decade, inconsistent standards for road roughness have led to varying conditions across different regions. This inconsistency has created maintenance challenges, including increased public complaints about unsafe conditions. To address these challenges, this study proposed a practical method for measuring both roughness and hazardous points on bicycle lanes using a smartphone’s 3-axis accelerometer and GPS sensors.

- Bicycle Road Roughness: The roughness of bicycle lanes was evaluated using a smartphone’s three-axis accelerometer, with a focus on the vertical (z-axis) acceleration. The absolute mean value of vertical acceleration, adjusted by a speed correction factor, was used to calculate the Bicycle Road Roughness Index (BRI). Field tests indicated that BRI values ranged from 0.2 to 0.8, with sections exceeding 0.5 being classified as hazardous, significantly reducing riding comfort.
- Risk Assessment: Bump impact tests were conducted using different fault sizes (10 mm, 20 mm, 30 mm) and bicycle approach speeds (15 km/h, 20 km/h, 30 km/h). Nine scenarios were tested, with 50 repetitions each. Results showed that as both bump size and approach speed increased, the Faulting Impact Index (FII) rose proportionally. For instance, at a speed of 25 km/h, a 10 mm bump resulted in an FII value of 80 m/s^2^, while bumps larger than 30 mm caused the FII to sharply exceed 130 m/s^2^.
- FII Calculation: The FII was derived by aggregating acceleration changes across the x, y, and z axes, offering a quantitative measure of the shock experienced by cyclists. This method provides a reliable way to identify and prioritize hazardous points based on scientific criteria, facilitating targeted road maintenance.
- Smartphone-Based Measurement Method: The smartphone-based method proposed is cost-effective, simple to implement, and ideal for use in areas where specialized equipment is unavailable. By attaching a smartphone to a bicycle, real-time road condition data can be easily collected, enabling road administrators to efficiently identify areas needing maintenance. The proposed method can also be used to evaluate newly constructed bicycle lanes, ensuring they adhere to safety and comfort standards.

In conclusion, this study provides a practical, quantitative approach to assessing bicycle lane roughness and hazard levels, offering a solution that can improve the management and maintenance of bicycle infrastructure. The method’s low cost and simplicity make it accessible for use by road administrators in the field. As more big data systems and a wider range of user data become available, this approach could be further refined to develop predictive models for managing and enhancing bicycle transportation networks, ultimately improving their safety and overall quality. The BRI used in this study was designed with practical field application in mind, prioritizing simplicity and compatibility with smartphone sensor data. By using the mean absolute value of vertical acceleration, the index reflects the dynamic disturbance experienced by the cyclist in a physically meaningful way, although it is not expressed in traditional energy- or frequency-based terms. To address the inherent speed dependence of acceleration-based metrics, a linear correction model was introduced and applied consistently across all measurements. Nonetheless, we recognize that future refinements could enhance the physical interpretability of the index by incorporating root-mean-square (RMS) acceleration, vibration energy formulations, or frequency-domain analysis techniques. These developments may help establish a stronger theoretical foundation while preserving the model’s usability for infrastructure monitoring and cyclist safety applications. Although the BRI is tailored for non-motorized pathways and differs in methodology from vehicle-based indices such as the IRI, establishing a correlation between the two could enhance the practical adoption of BRI in infrastructure management. Future work may explore comparative analyses across shared-use or mixed-traffic paths where both metrics can be measured simultaneously, enabling a more rigorous standardization of BRI thresholds in line with engineering practice.

In future studies, the use of electric bicycles (e-bikes) equipped with cruise control or speed-assist features could offer a more stable platform for maintaining constant speeds during data collection. This may enhance the precision of acceleration-based measurements and further reduce variability caused by human pedaling behavior.

## Figures and Tables

**Table 1:** Korea’s guidelines for the installation and management of bicycle facilities: pavement roughness service standards.

Roughness Grade	Criteria
A	A road that provides a high level of ride comfort with a newly constructed pavement condition, showing no signs of deterioration
B	A road with high ride quality, some signs of surface deterioration but still providing a smooth ride, though not as a newly constructed road
C	A road with visible cracks or surface wear, but re-paved, allowing for high-speed travel despite a decrease in ride comfort
D	A road with significant surface deterioration that negatively impacts travel speed
E	A road where normal travel is not possible due to severe deterioration

**Table 2:** Experimental setup method for measuring hazards on bicycle road.

Category	Experiment	Height	Bicycle Speed
Step difference (Upper)	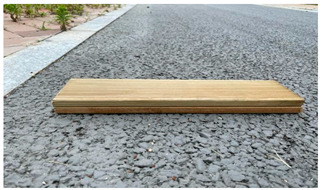	1 cm 2 cm 3 cm 4 cm	15 km/h 20 km/h 25 km/h
Pothole/Sinkhole (Downward)	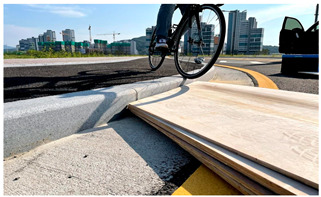	1 cm 2 cm 3 cm 4 cm	15 km/h 20 km/h 25 km/h

**Table 3:** Summarize method of roughness and risk assessment test in bicycle road.

Roughness Test		Risk Assessment Test	
**Category**	Contents	Category	Contents
Experiment Objective	Bicycle road surface roughness by expected service level	Experiment Objective	Measurement of FII according to bump height and approach speed
Road type and Pavement type	Level A (excellent): Exclusive bicycle road, asphalt pavement Level B (good): Separated bicycle-pedestrian shared road, asphalt pavement Level C (fair): Exclusive bicycle road, concrete pavement Level D (poor): Shared automobile-bicycle road, concrete pavement Level E (Very Poor): Shared automobile-bicycle road, concrete pavement	Hazard type	Wooden square timber k (used for upward step measurement)
Road surface Characteristics	Service A (excellent): Newly constructed bicycle road Service Level B (good): Excellent roughness condition Service Level C (fair): Partial roughness degradation due to labeling, etc. Service Level D (poor): Occurrence of labeling, spalling, cracks, etc. Service Level E (very poor): Excessive anti-slip pavement causing difficulty in riding	Scenario	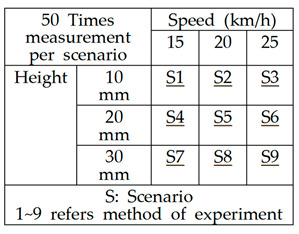
Experimental Conditions	Measurement device: Road bicycle (wheel size 700 C, tire pressure 110 psi) Measurement smart device: Samsung Galaxy Note Sensor type: 3-axis accelerometer and GPS sensor Measurement interval: 0.1 s Measurement content: Z-axis acceleration	Experimental Conditions	-Measurement device: Road bicycle (wheel size 700 C, tire pressure 110 psi) -Measurement smart device: Samsung Galaxy Note Sensor type: 3-axis accelerometer and GPS sensor -Measurement interval: 0.01 s -Measurement content: FII (sum of 3-axis accelerations at the moment of maximum combined x, y, z acceleration during impact)
Experimental Method	Mount smartphone using fixed equipment on the measuring device Measure 3-axis acceleration Ride the target section at 25 km/h and end measurement	Experimental Method	-Smartphone using fixed equipment on the measuring device -Run the 3-axis accelerometer application -Impact the wooden square timber of specified bump height while maintaining the designated speed

**Table 4:** Comparison of BRI statistics for expected service levels using sensors.

Category	Scenario 1	Scenario 2	Scenario 3	Scenario 4	Scenario 5
Mean	3.040162	2.28489	6.774157	14.4497	22.58115
Standard error	0.29189	0.17931	0.50507	0.94367	2.14620
Median	2.2535	2.0793	5.9453	12.9231	12.4256
Standard deviation	3.14374	1.93124	4.71104	10.9645	23.9952
Variance	9.8831	3.7297	−0.8017	120.2209	575.7736
Kurtosis	2.5369	−0.1688	0.4969	2.5284	−0.0706
Skewness	18.7104	0.7899	0.4969	1.3254	1.0836
Range	0.0176	7.5635	17.8878	59.7315	86.7817
Minimum	0.0176	0.0023	0.0554	0.2447	0.2093
Maximum	18.7280	7.5658	17.9432	59.9762	86.991

**Table 5:** Comparison of BRI statistics for expected service levels using sensors.

Category	Results								
Target speed(km/h)	5.0	10.0	15.0	20.0	25.0	30.0	35.0	40.0	45.0
Actual speed(km/h)	5.6	10.6	14.7	20.4	24.6	28.6	34.5	37.3	45.0
Driver error(km/h)	0.6	0.6	0.3	0.4	0.4	1.4	0.5	2.7	0

**Table 6:** BRI calibration factors with different bicycle speed.

$\bar{v_{ij}}$ (km/h)	${scf}_{ij}$	$\bar{v_{ij}}$ (km/h)	${scf}_{ij}$	$\bar{v_{ij}}$ (km/h)	${scf}_{ij}$	$\bar{v_{ij}}$ (km/h)	${scf}_{ij}$
6	4.25	16	1.57	26	0.96	36	0.69
7	3.63	17	1.47	27	0.93	37	0.67
8	3.16	18	1.39	28	0.89	38	0.66
9	2.81	19	1.32	29	0.86	39	0.64
10	2.52	20	1.25	30	0.83	40	0.62
11	2.29	21	1.19	31	0.81	41	0.61
12	2.10	22	1.14	32	0.78	42	0.59
13	1.93	23	1.09	33	0.76	-	-
14	1.79	24	1.04	34	0.73	-	-
15	1.67	25	1.00	35	0.71	-	-

**Table 7:** BRI results of statistical with different bicycle speed.

Category	15 km/h			20 km/h			25 km/h		
	S1	S4	S7	S2	S5	S8	S3	S6	S9
Maximum	78.7	97.6	119.9	109.0	112.9	137.0	96.9	124.4	130.3
Minimum	31.4	43.4	48.2	45.7	48.9	67.0	52.3	57.6	67.5
Average	54.8	71.9	98.8	69.1	87.2	103.4	79.9	101.8	116.6
Median	53.6	72.7	104.4	69.7	87.6	103.2	80.4	101.4	118.8
Standard Deviation	12.2	14.6	17.6	14.6	14.3	13.0	9.6	16.7	12.9

S: Scenario (the specific method of scenario is described in Table 3).

**Table 8:** Average FII based on the driving speed and the size of the step difference.

Bump Height	Driving Speed 15 km/h	Driving Speed 20 km/h	Driving Speed 30 km/h
10 mm	54.8	69.1	79.9
20 mm	71.9	87.2	101.8
30 mm	98.8	103.4	111.6

## Data Availability

The bicycle traffic volume and speed data from the tracking of cycling were provided by Strava application and are available online: https://www.strava.com. And detailed value of roughness is measured based on self-developed applications.( accessed on 20 October 2022.).
